# The Effect of IFT80 Deficiency in Osteocytes on Orthodontic Loading-Induced and Physiologic Bone Remodeling: In Vivo Study

**DOI:** 10.3390/life12081147

**Published:** 2022-07-29

**Authors:** Hyeran Helen Jeon, Jessica Kang, Jiahui (Madelaine) Li, Douglas Kim, Gongsheng Yuan, Nicolette Almer, Min Liu, Shuying Yang

**Affiliations:** 1 Department of Orthodontics, School of Dental Medicine, University of Pennsylvania, Philadelphia, PA 19104, USA; jesskang@upenn.edu (J.K.); jiahuili@upenn.edu (J.L.); douglas.w.kim@gmail.com (D.K.); nalmer@upenn.edu (N.A.); 2 Department of Basic and Translational Sciences, School of Dental Medicine, University of Pennsylvania, Philadelphia, PA 19104, USA; gsyuan@upenn.edu; 3 Department of Periodontics, School of Dental Medicine, University of Pennsylvania, Philadelphia, PA 19104, USA; minliu@upenn.edu; 4 The Penn Center for Musculoskeletal Disorders, School of Dental Medicine, University of Pennsylvania, Philadelphia, PA 19104, USA; 5 Center for Innovation & Precision Dentistry, School of Dental Medicine, School of Engineering and Applied Sciences, University of Pennsylvania, Philadelphia, PA 19104, USA

**Keywords:** IFT80, cilia, osteocyte, orthodontic tooth movement, bone remodeling, sclerostin, RANKL

## Abstract

Osteocytes are the main mechanosensory cells during orthodontic and physiologic bone remodeling. However, the question of how osteocytes transmit mechanical stimuli to biological responses remains largely unanswered. Intraflagellar transport (IFT) proteins are important for the formation and function of cilia, which are proposed to be mechanical sensors in osteocytes. In particular, IFT80 is highly expressed in mouse skulls and essential for ciliogenesis. This study aims to investigate the short- and long-term effects of IFT80 deletion in osteocytes on orthodontic bone remodeling and physiological bone remodeling in response to masticatory force. We examined 10-week-old experimental DMP1 CRE^+^.IFT80^f/f^ and littermate control DMP1 CRE^−^.IFT80^f/f^ mice. After 5 and 12 days of orthodontic force loading, the orthodontic tooth movement distance and bone parameters were evaluated using microCT. Osteoclast formation was assessed using TRAP-stained paraffin sections. The expression of sclerostin and RANKL was examined using immunofluorescence stain. We found that the deletion of IFT80 in osteocytes did not significantly impact either orthodontic or physiologic bone remodeling, as demonstrated by similar OTM distances, osteoclast numbers, bone volume fractions (bone volume/total volume), bone mineral densities, and the expressions of sclerostin and RANKL. Our findings suggest that there are other possible mechanosensory systems in osteocytes and anatomic limitations to cilia deflection in osteocytes in vivo.

## 1. Introduction

Orthodontic tooth movement (OTM) is an effective model for studying the mechanical loading-induced bone remodeling [1]. Mechanical force application to a tooth initiates the remodeling of periodontal ligament (PDL) and alveolar bone around the tooth. Osteoclasts resorb bones under compression, while osteoblasts produce new bones in response to tensional force. Osteocytes, terminally differentiated osteoblasts, are embedded within the mineralized bone matrix and individually reside in lacunae. Osteocytes are the key mechanosensory cells in bones, comprising 90–95% of all bone cells, and they coordinate the functions of both osteoclasts and osteoblasts during bone remodeling [2,3,4]. Furthermore, osteocytes are one of the most important sources of sclerostin and RANKL in alveolar bone remodeling during OTM [4,5,6,7]. RANKL is a key growth factor for stimulating osteoclastogenesis [8]. Sclerostin is closely related to osteocyte mechanotransduction by its effect on the Wnt/β-catenin pathway, which inhibits new bone formation [1,3,9,10]. Yet, it is not entirely understood how osteocytes sense mechanical loading during OTM and regulate bone remodeling accordingly.

The primary cilium is located on the surface of most vertebrate cells. This hair-like nonmotile structure serves as both a mechanosensor and chemosensor in various tissues, including bones, cartilage, and kidneys [11,12,13,14,15,16,17,18,19,20]. Primary cilia in osteoblasts and osteocytes can deflect during fluid flow and regulate the expression of osteopontin, prostaglandin E_2_ (PGE_2_), cyclooxygenase 2 (COX2), and RANKL [21,22], thereby controlling osteogenic and osteoclastic responses to dynamic fluid flow. In addition, primary cilia positively correlate with osteocyte mechanosensitivity in vitro, and osteocyte mechanosensitivity increases with cilium elongation [23]. Cilia and cilia-related proteins are widely involved in mechanical force-induced osteogenesis, while cilia on osteoblasts and osteocytes indirectly regulate osteoclastogenesis by affecting the ratio of osteoprotegerin (OPG) to RANKL in vitro [21]. Intraflagellar transport (IFT) proteins are important for cilia development and bone remodeling [24]. IFT80 is a complex B protein of the IFT family and greatly expressed in mouse skulls, long bones, and during osteoblast differentiation [24,25]. Deletion of IFT80 causes either the loss or shortening of the cilia and can block osteoblast marker expression, thus substantially inhibiting alkaline phosphatase (ALP) activity and mineralization [24]. To date, the role of IFT80 in osteocytes has not been investigated in vivo and its functions during OTM remain unknown [23].

The aim of this study is to examine the short- and long-term effects of IFT80 deletion in osteocytes during OTM and physiologic bone remodeling, in response to mastication using transgenic experimental DMP1 CRE^+^.IFT80^f/f^ and littermate control DMP1 CRE^−^.IFT80^f/f^ mice. 

## 2. Materials and Methods

### 2.1. Transgenic Mice

The University of Pennsylvania Institutional Animal Care and Use Committee approved the protocol (protocol number: 806005) on 1 July 2019 and experiments were carried out, adhering to the guidelines and regulations. Dentin matrix protein 1 (Dmp1) plays a major role in controlling osteocyte formation, maturation, phosphate homeostasis, and mineralization [26,27]. DMP1 CRE mice express Cre recombinase under a control element of the Dmp1 promoter, so that expression is restricted to osteocytes [27,28]. An IFT80^f/f^ mouse model with two LoxP sites flanking exon 6 of IFT80 was generated, as previously described [29]. Mice harboring an IFT80 allele (IFT80^f/f^) were bred with DMP1 Cre mice to generate experimental DMP1 CRE^+^.IFT80^f/f^ and control DMP1 CRE^−^.IFT80^f/f^ mice. We used 10-week-old DMP1 CRE.IFT80^f/f^ mice in this experiment. Each cage contained two to five mice housed with a 14-h light/10-h dark cycle in standard laboratory conditions. The mice were provided soft food and water ad libitum for the duration of the study.

### 2.2. Genotyping

Genomic DNA from mouse ear tissues or tails was analyzed by PCR, following the manufacturer’s instruction (Jackson Laboratory, Bar Harbor, ME, USA). Primers utilized for genotyping were (for DMP1-cre) 5-TTG CCT TTC TCT CCA CAG GT-3 (transgene forward), 5-CAT GTC CAT CAG GTT CTT GC-3 (transgene reverse), 5-CTA GGC CAC AGA ATT GAA AGA TCT-3 (internal positive control forward), and 5-GTA GGT GGA AAT TCT AGC ATC ATC C-3 (internal positive control reverse); (for IFT80^f/f^) 5- TGTGAGGCCAGCCCGAGTTA-3 (forward) and 5-GCCTGAGCTACAGAGAGACCCCACG-3 (reverse) (Figure S1).

### 2.3. OTM Model

OTM experiments were carried out as previously described [30,31]. The 10 DMP1 CRE^+^.IFT80^f/f^ mice and 10 DMP1 CRE^−^.IFT80^f/f^ mice were randomly assigned into day 5 and 12 groups (*n* = 5 for each group, *n* = 20 in total) [32,33]. Two males and three females were assigned for the day 5 group, and three males and two females were assigned for the day 12 group. A 0.006 × 0.030 nickel-titanium coil spring was placed between the maxillary incisors and right 1st molar with ligature wires and light-cured dental composite resin under anesthesia using intraperitoneal injection of ketamine (80 mg/kg), xylazine (5 mg/kg), and acepromazine (1 mg/kg) [30]. We used a force level of 35 g, which is considered the appropriate force level to study OTM in mice without any side effects [34]. No reactivation was carried out during the study. The left side served as a control with no orthodontic force placed on it. The mice were sacrificed after OTM loading for 5 or 12 days to examine the early and late effect of IFT80 deletion in osteocytes on OTM, respectively. For day 5, we focused on the cytokine expression and osteoclast formation. For day 12, we stressed on bony responses, including the OTM distance and bone parameters, such as BV/TV and BMD [30,31]. 

### 2.4. MicroCT

The maxillary molars and the adjoining alveolar bone were fixed for 24 h at 4 °C in 4% paraformaldehyde in PBS. Micro-CT (MicroCT35; SCANCO Medical, Bassersdorf, Switzerland) was used to scan every sample with a 20 µm isotropic voxel with the following settings: 145 µA, 55 kVp, and an integration time of 200 ms. The microCT images were converted to DICOM files and analyzed using OsiriX (Pixmeo SARL, Bernex, Switzerland). The smallest distance between the maxillary right 1st and 2nd molar crowns was calculated. We used a 250 × 250 × 250 µm^3^ ROI for the bone parameter measurements. For the tension side, we examined the coronal third of the mesiobuccal roots; for the compression side, we measured on the coronal third of the distobuccal roots of maxillary 1st molars [31,35]. On both sides, we measured the bone volume fractions (bone volume/total volume, BV/TV), ratio of the segmented bone volume to the total volume of the region of interest, and bone mineral densities (BMD), respectively. Physiological bone remodeling, in response to masticatory force, was assessed at the furcation area of the maxillary and mandibular left 1st molar, in order to examine the long-term effect of IFT80 deletion in osteocytes [36,37]. 

### 2.5. Histology and TRAP Stain

Samples were decalcified in a solution of 10% EDTA with constant agitation over the course of 5 weeks at 4 °C. The samples were processed in paraffin or frozen blocks. Tartrate-resistant acid phosphatase (TRAP) stain was carried out using 4-μm paraffin sections, according to the manufacturer’s protocol (Sigma-Aldrich, Saint Louis, MO, USA). The number of multinucleated TRAP-positive cells was counted along the bony surfaces and divided by the length on the compression and tension sides of the distobuccal root of the maxillary 1st molar using 10× and 20× objectives. The NIS-elements software (Nikon, Melville, NY, USA) was used to analyze the TRAP-stained section images. 

### 2.6. Immunofluorescence Stain

Antigen retrieval was carried out in 10 mM of citric acid, pH 6.0, at 100 °C for one hour. Sections were incubated with primary antibody to IFT80 (PAB27850; Abnova, Taipei, Taiwan), acetylated α-tubulin (T6793; Sigma Aldrich, St. Louis, MO, USA), RANKL (ab216484; Abcam, Cambridge, MA, USA), and sclerostin (AF1589; R&D Systems, Minneapolis, MN, USA) overnight at 4 °C, as well as the matched negative control (I-1000 or I-5000; Vector Laboratories, Inc., Newark, CA, USA). HRP-conjugated secondary antibodies (705-035-147 or 111-035-144; Jackson ImmunoResearch Laboratories, Inc., West Grove, PA, USA), Alexa Fluor™ 647 tyramide reagent (B40958; ThermoFisher, Waltham, MA, USA), or Alexa Fluor™ 488 tyramide reagent (B40953) and DAPI mounting media (Sigma-Aldrich, St. Louis, MO, USA) were used. Four to six images per sample were taken at 40× objectives and assessed with NIS-Element software (Nikon) to examine the numbers of immunopositive osteocytes, divided by the area or total osteocytes on the compression side of the distobuccal root of maxillary 1st molar. Images were examined by a double-blinded examiner, and the results were confirmed with a second examiner. For all experiments, capture times were determined, so that the control IgG had no immunofluorescence.

### 2.7. Real-Time PCR

TRIzol reagent (Sigma–Aldrich, US) was used to isolate RNA from the long bone tissues (femurs and tibiae), according to the manufacturer’s protocol. A total of 1 µg of RNA was then reverse-transcribed using a reverse-transcription kit (Takara, Japan) into cDNA. Real-time PCR was carried out with a reaction solution containing primers, the cDNA template, and SYBR green PCR master mix (Bimake, Houston, TX, USA). The sequences of the real-time PCR primers are as follows: IFT80, (forward) 5′-AGTTATTTGCCGTTGGATCG-3′ and (reverse) 5′-CCTGCATGGTCCTTCTCTTC-3′; GAPDH, (forward) 5′-AGGTCGGTGTGAACGGATTTG-3′ and (reverse) 5′-TGTAGACCATGTAGTTGAGGTCA-3′.

### 2.8. Western Blot

Long bones from the DMP1 CRE^+^.IFT80^f/f^ and DMP1 CRE^−^.IFT80^f/f^ mice were homogenized with RIPA buffer (50 mM Tris, 150 mM NaCl, 1% Triton X-100, 0.1% SDS, and 1% sodium deoxycholate) containing PIC (Sigma Aldrich, US) on ice. Equal amounts of protein (20 μg) were denatured in SDS and separated in 10% SDS–PAGE gels. Proteins were transferred to nitrocellulose membranes in transfer buffer containing 20% methanol. The membranes were blocked with 5% skim milk, incubated with primary antibodies (β-Actin, 1:3000, Santa Cruz, No. sc-47778 HRP; IFT80, 1:1000, Proteintech, No. 25230-1-AP) overnight at 4 °C, and then incubated with horseradish peroxidase (HRP)-conjugated second antibody (1:5000, Jackson ImmunoResearch, West Grove, PA, USA) at 20–22 °C room temperature for 1 h. Signals were analyzed using an ECL Western blotting system (Bio-Rad Laboratories, Hercules, CA, USA). 

### 2.9. Statistics

Statistical analysis was performed using 2-tailed Student’s *t*-test to compare experimental and control mice. Differences across multiple groups were computed by ANOVA with Scheffe’s post hoc test. Results were reported as the mean ± SEM. *p* < 0.05 was considered statistically significant. 

## 3. Results

### 3.1. IFT80 Expression and Cilia Number Significantly Decreases in Osteocytes of DMP1 CRE^+^.IFT80^f/f^ Mice

**Figure 1:**
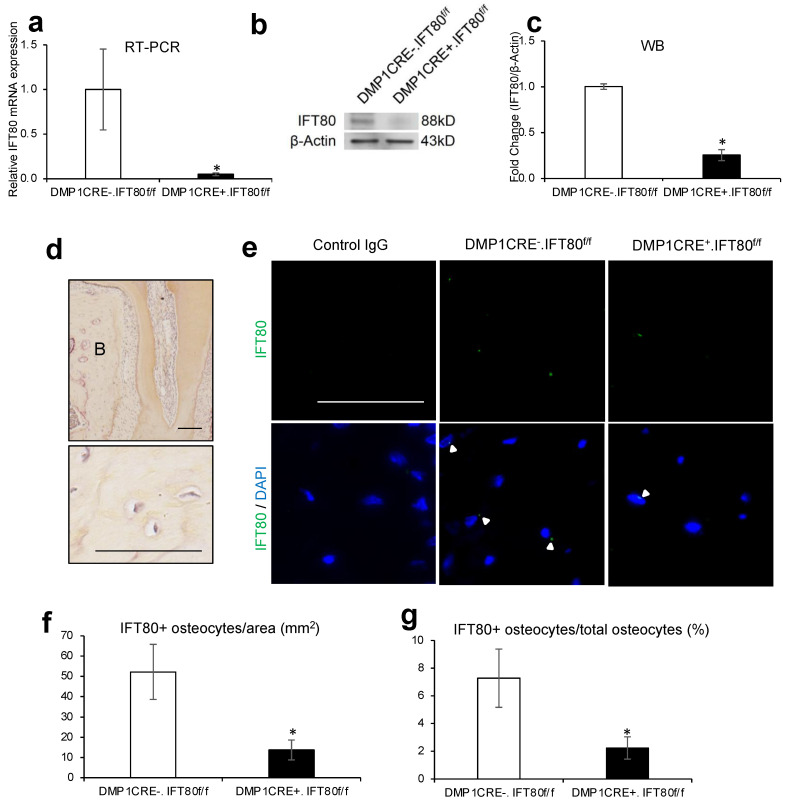

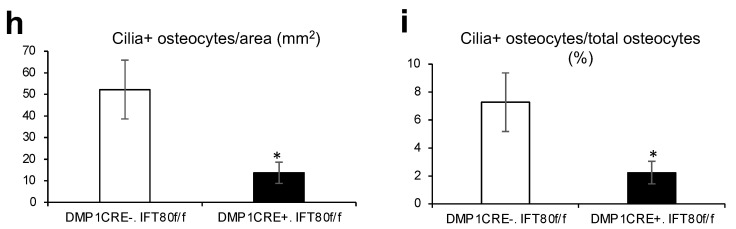
**IFT80 deletion in osteocytes.** (**a**–**c**) The expression levels of IFT80 mRNA and protein in the bone of 10 weeks old DMP1 CRE^−^.IFT80^f/f^ and DMP1 CRE^+^.IFT80^f/f^ mice, as measured by qRT-PCR (*n* = 3) and western blot (*n* = 3). *, *p* < 0.05 versus control mice group. (**d**) Histologic images of the distobuccal root of the maxillary left 1st molar at 10× (upper) and 40× (lower). B: bone. Bar, 100 μm (upper) and 50 μm (lower). (**e**) IFT80 expression was examined on the furcation area and mesial side of the distobuccal root of the maxillary left 1st molar using an IFT80 antibody (40×). The arrowheads represent the positive IFT80 expression in osteocytes. Bar, 50 μm. (**f**) The number of IFT80 immunopositive cells per area. (**g**) The percentage of IFT80 immunopositive cells per total osteocytes (*n* = 7). (**h**) The number of acetylated α-tubulin immunopositive cells per area. (**i**) The percentage of acetylated α-tubulin immunopositive cells per total osteocytes (*n* = 7). *, *p* < 0.05 versus control mice group. The 2-tailed student’s *t*-tests were performed.

General physical appearance looked similar. The average weights at 10 weeks for DMP1 CRE^−^.IFT80^f/f^ and DMP1 CRE^+^.IFT80^f/f^ mice were 24.6 ± 2.67 g and 24.75 ± 3.66 g, respectively (*p* = 0.86). Real-time PCR and western blot analysis demonstrated a 94.9% decrease (*p* = 0.04) and 74.5% decrease (*p* = 0.002), respectively, in IFT80 expression in the long bone of DMP1 CRE^+^.IFT80^f/f^ mice, compared with the control DMP1 CRE^−^.IFT80^f/f^ mice (Figure 1a–c). 

To further examine the IFT80 expression level in osteocytes, the paraffin sections of the furcation area and mesial side of distobuccal root of maxillary left 1st molar were used for immunofluorescence stain of IFT80 protein (Figure 1d,e). Osteocytes are the most abundant cells in bones, which are identified as a single cell residing in small lacuna in the calcified matrix. The cell body varies in size from 5–20 μm in diameter, with a cell-to-cell distance between 20–30 μm [38,39]. Based on the above features, we could easily identify the osteocytes in the bone sections. The result showed that IFT80 expression was significantly reduced in osteocytes of experimental DMP1 CRE^+^.IFT80^f/f^ mice, compared to the control group (Figure 1f,g). The value of IFT80-immunopositive osteocytes divided by area decreased by 73.8% (*p* = 0.02), and the value of IFT80-immunopositive osteocytes divided by total osteocytes number was reduced by 69.3% (*p* = 0.04) in experimental DMP1 CRE^+^.IFT80^f/f^ mice, in comparison to the control mice. The ciliated osteocytes, indicated by the number of acetylated α-tubulin-immunopositive osteocytes divided by area, decreased by 58.1% (*p* = 0.02), and the value of ciliated osteocytes divided by total osteocytes number was reduced by 61.8% (*p* = 0.03) in experimental DMP1 CRE^+^.IFT80^f/f^ mice, in comparison to the control mice (Figure 1h,i). We had technical difficulty measuring the cilia length, due to very short cilia in osteocytes in vivo. 

### 3.2. IFT80 Deletion in Osteocytes Does Not Affect Orthodontic Tooth Movement

**Figure 2:**
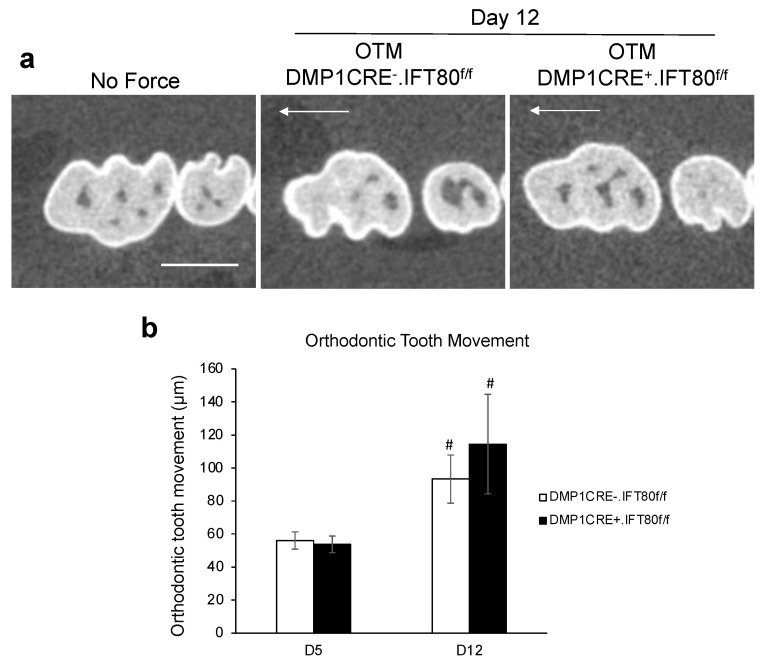
**Measurement of the Orthodontic Tooth Movement (OTM).** (**a**) OTM was measured as the smallest distance between the maxillary right 1st and 2nd molar crowns. Bar, 1 mm. Arrow represents the direction of tooth movement. (**b**) Lineage-specific deletion of IFT80 in osteocytes did not significantly impact the OTM distance after 5 or 12 days of orthodontic loading. Each in vivo value is the mean ± SEM for *n* = 5 mice per group. #, *p* < 0.05 versus day 5 matched mice group. An ANOVA with Scheffe’s post hoc test was performed.

OTM distance was assessed in both experimental DMP1 CRE^+^.IFT80^f/f^ and control DMP1 CRE^−^.IFT80^f/f^ mice by measuring the smallest distance from the maxillary right 1st to 2nd molar crowns using microCT (Figure 2). Teeth in control mice and experimental mice moved 56.1 ± 5.21 and 53.72 ± 5.05 µm on day 5 and 93.29 ± 14.53 and 117.15 ± 27.44 µm on day 12. There was a significant difference between day 5 and 12 matched groups (*p* = 0.048 for DMP1 CRE^−^.IFT80^f/f^ mice and *p* = 0.046 for DMP1 CRE^+^.IFT80^f/f^ mice). However, no difference was observed between experimental and control groups under OTM loading (*p* = 0.78 for day 5 and *p* = 0.48 for day 12).

### 3.3. Deletion of IFT80 in Osteocytes Does Not Affect Bone Remodeling during OTM and Physiologic Bone Remodeling

**Figure 3:**
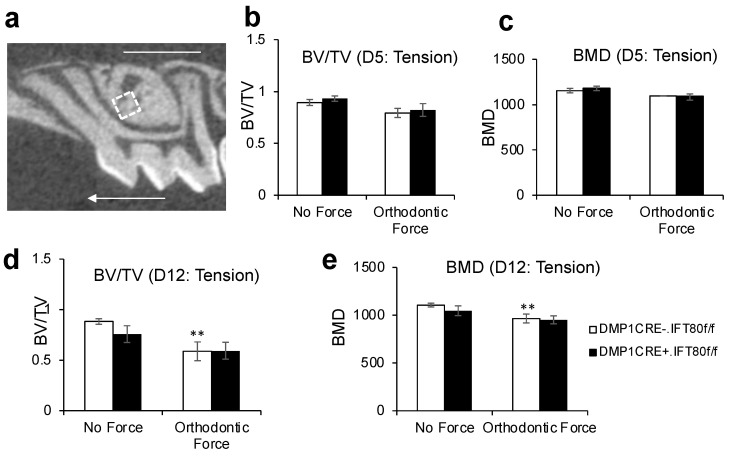

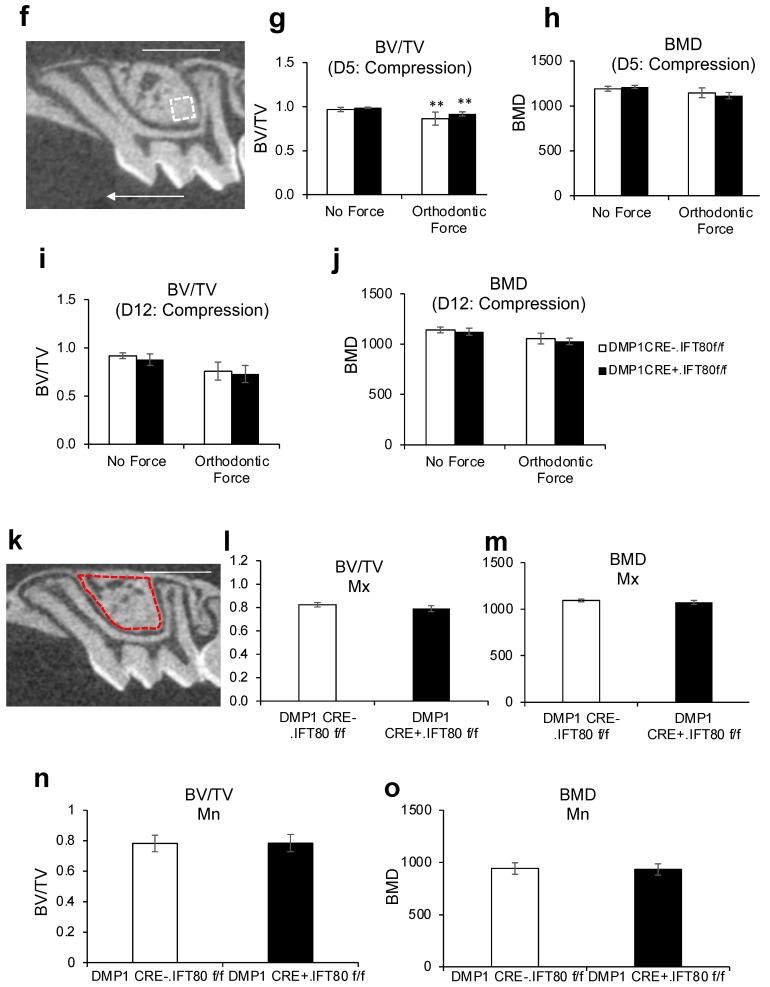
**MicroCT Analysis on Tension and Compression side during OTM and Physiologic Bone Remodeling.** (**a**) Bone volume fraction (BV/TV) and bone mineral density (BMD) were measured on the cervical third of the tension side of the mesiobuccal root of the maxillary right 1st molar using a ROI (white square) of 250 × 250 × 250 µm^3^. Arrow represents the direction of tooth movement. (**b**,**c**) BV/TV and BMD on day 5. (**d**,**e**) BV/TV and BMD on day 12. (**f**) BV/TV and BMD were measured on the cervical third of the compression side of the distobuccal root of the maxillary right 1st molar using a ROI of 250 × 250 × 250 µm^3^. (**g**,**h**) BV/TV and BMD on day 5. (**i**,**j**) BV/TV and BMD on day 12. **, *p* < 0.05 versus “No Force” matched mice group. Each in vivo value is the mean ± SEM for *n* = 5 mice per group. (**k**) BV/TV and BMD were measured to examine physiologic bone remodeling in response to mastication at the furcation area of the unloaded maxillary and mandibular left 1st molar (red broken line). (**l**) BV/TV (Mx: maxilla). (**m**) BMD (Mx). (**n**) BV/TV (Mn: mandible). (**o**) BMD (Mn). Each in vivo value is the mean ± SEM for *n* = 10 mice per group. The ANOVA with Scheffe’s post hoc test (**b**–**e**,**g**–**j**) and 2-tailed student’s *t*-tests (**l**–**o**) were performed.

To determine whether deletion of IFT80 in osteocytes affects maxillary bone remodeling around the tooth under OTM, we examined BV/TV and BMD on the tension area of the coronal third of the mesiobuccal root of the maxillary 1st molar using a region of interest (ROI) size of 250 × 250 × 250 µm^3^ (Figure 3a). The result from MicroCT analysis showed similar values of BV/TV and BMD in all groups on day 5 (*p* = 0.15–0.71 in Figure 3b and *p* = 0.15–0.86 in Figure 3c). On day 12, the control mice showed a 29% reduction (0.63 ± 0.08) in BV/TV under orthodontic loading, compared to the unloaded side (0.89 ± 0.03) (*p* = 0.002, Figure 3d), and 15% decrease (982.34 ±37.63 HA/ccm) in BMD, compared to the unloaded side (1155.03 ± 25.15 HA/ccm) (*p* = 0.002, Figure 3e). However, both the experimental and control groups showed a decrease in BV/TV and BMD on day 12, and no significant difference between the DMP1 CRE^+^.IFT80^f/f^ and DMP1 CRE^−^.IFT80^f/f^ mice was observed (*p* = 0.74 in Figure 3d and *p* = 0.56 in Figure 3e). In addition, we examined the BV/TV and BMD on the compression area of the coronal third of the distobuccal root of the maxillary 1st molar (Figure 3f–j). We found a slight decrease in the BV/TV and BMD after orthodontic force loading in both the experimental and control mice, which was statistically insignificant (*p* = 0.44 in Figure 3i and *p* = 0.45 in Figure 3j). 

Alveolar bone provides structural support against masticatory forces, and signals from alveolar bone bending during normal chewing are important for bone maintenance around teeth. In order to examine the effects of IFT80 deletion on physiological bone remodeling in response to masticatory forces, we also calculated BV/TV and BMD in the furcation areas of the unloaded maxillary and mandibular left 1st molars (Figure 3k–o). The results demonstrated that no statistically significant differences in any bone parameters for physiological bone remodeling in response to chewing were observed between the experimental DMP1 CRE^+^.IFT80^f/f^ and control DMP1 CRE^−^.IFT80^f/f^ mice (*p* = 0.80 for Figure 3l, *p* = 0.96 for Figure 3m, *p* = 0.97 for Figure 3n, and *p* = 0.90 for Figure 3o).

### 3.4. Loss of IFT80 in Osteocytes Slightly Affects Osteoclast Formation during OTM but without Statistical Difference

**Figure 4:**
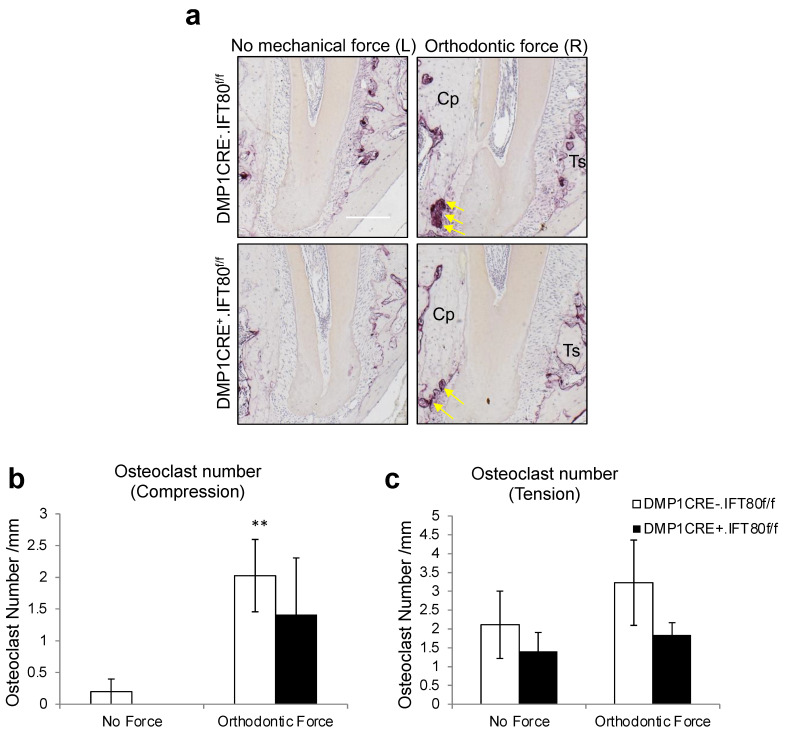
**Osteoclast Formation.** (**a**) The number of osteoclasts was examined on the compression side of the distobuccal root of the maxillary right 1st molar on day 5 (10×). Bar, 100 μm. Cp, compression side; Ts: tension side. Yellow arrows indicate TRAP-positive multinucleated osteoclasts along the bony surface. (**b**) Osteoclast formation on compression side. (**c**) Osteoclast formation on tension side. Each in vivo value is the mean ± SEM for *n* = 5 mice per group. **, *p* < 0.05 versus “No Force” matched mice group. The ANOVA with Scheffe’s post hoc test was performed.

Osteoclast numbers were examined on the compression and tension sides of the maxillary 1st molar distobuccal root using the TRAP-stained samples (Figure 4a). Osteoclasts numbers greatly increased on the compression side with the orthodontic force application in the control mice (*p* = 0.03) and increased less in the experimental mice (*p* = 0.22, Figure 4b). There were 30% less osteoclasts in the experimental DMP1 CRE^+^.IFT80^f/f^ group (1.41 ± 0.89), compared to the control DMP1 CRE^−^.IFT80^f/f^ group (2.03 ± 0.57) on the compression side, and 43% less in the experimental group (1.84 ± 0.33), compared to the control group (3.23 ± 1.13) on the tension side (Figure 4b,c). However, both differences between DMP1 CRE^+^.IFT80^f/f^ and DMP1 CRE^−^.IFT80^f/f^ mice were statistically insignificant (*p* = 0.22, Figure 4b and *p* = 0.48, Figure 4c). 

### 3.5. Ablation of IFT80 in Osteocytes Does Not Alter Expression Levels of Sclerostin and RANKL

**Figure 5:**
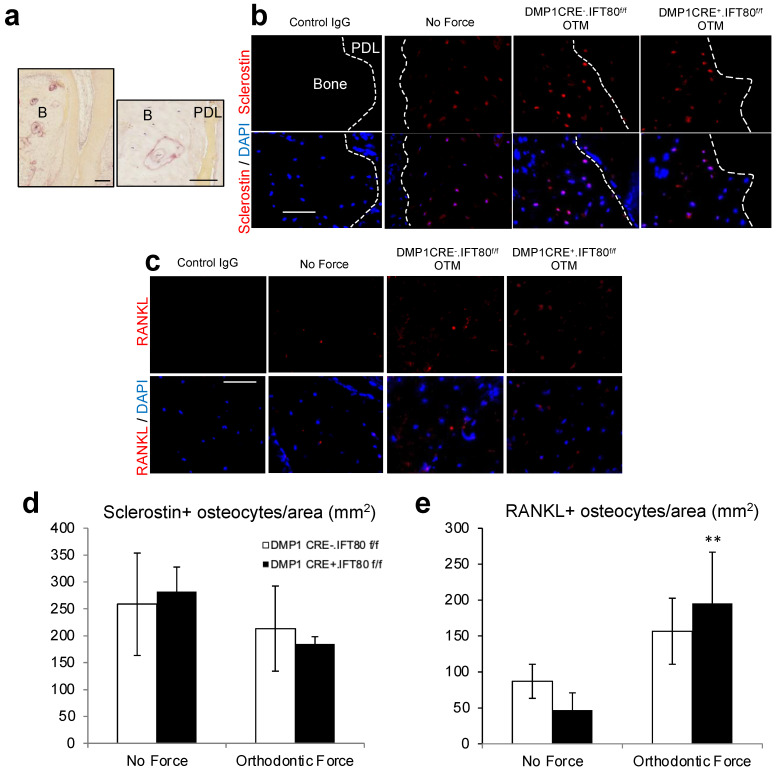
**Sclerostin and RANKL Immunofluorescence Stain.** (**a**) Histologic images of the distobuccal root of the maxillary right 1st molar at 10× (left) and 40× (right). B: bone. PDL: periodontal ligament space. Bar, 100 μm (left) and 50 μm (right). (**b**) Sclerostin expression was examined on the compression side of the distobuccal root of the maxillary right 1st molar using a sclerostin antibody (40×). Bar, 50 μm. (**c**) RANKL expression was examined on the compression side of the distobuccal root of the maxillary right 1st molar using a RANKL antibody (40×). Bar, 50 μm. (**d**) The number of sclerostin immunopositive osteocytes per area were examined on day 5. (**e**) The number of RANKL immunopositive osteocytes per area were examined on day 5 on the compression side of the distobuccal root of the maxillary right 1st molar. Each in vivo value is the mean ± SEM for *n* = 5 mice per group. **, *p* < 0.05 versus “No Force” matched mice group. The ANOVA with Scheffe’s post hoc test was performed.

To further investigate whether a deficiency of IFT80 in the osteocytes affects the expression of sclerostin and RANKL on the compression side, we performed immunofluorescence stain for these two proteins (Figure 5a–e). After 5 days of orthodontic loading, there was no statistically significant difference between experimental DMP1 CRE^+^.IFT80^f/f^ and control DMP1 CRE^−^.IFT80^f/f^ mice in the number of sclerostin-positive osteocytes per area (*p* = 0.74, Figure 5b,d). Mechanical loading caused a 1.8–4.2-fold increase in the RANKL expression, compared with the unloaded side. However, there was no statistically significant difference in the sclerostin and RANKL expressions between the experimental and control groups (*p* = 0.39, Figure 5c,e).

## 4. Discussion

This is the first study to investigate the function of IFT80 in osteocytes under mechanical loading-induced and physiologic bone remodeling in vivo. Ablation of IFT80 in osteocytes did not show significant short- and long-term effects on alveolar bone remodeling during OTM and physiologic bone remodeling, as reflected by similar OTM distances, BV/TV, BMD, osteoclast formation, and the expression of sclerostin and RANKL between the experimental DMP1 CRE^+^.IFT80^f/f^ and control DMP1 CRE^−^.IFT80^f/f^ mice. There are three possible explanations for these results. First, previous studies reported the controversial role of primary cilia in osteocytes in vivo for mechanosensing, due to the anatomical limitations [4,21,22,40,41,42,43]. In vitro murine osteocytes have cilia that are 2–9 μm in length [21,22]. However, the pericellular space between the osteocyte and lacunae is less than 1μm [40], which is much smaller than the length of the cilia. This would make cilia deflection very difficult [21,41]. Supporting that, the in vivo primary cilia from the embedded osteocytes have a much shorter average lengths of 1.62–4 μm, in general [22,42]. In addition, the primary cilium is located on the cell body of each osteocyte, not the dendritic process. Considering that fluid flow induced by mechanical loading occurs around dendritic processes and mechanosensitivity in dendritic processes is higher than that of the cell bodies of osteocytes [43], the role of primary cilia in vivo in osteocytes remains under debate [4]. Secondly, even though primary cilia in osteocytes in vitro regulate flow shear stress, OTM loading is not the same type of mechanical loading. In addition, primary cilia in osteocytes may respond differently to different kinds of mechanical loadings [4,44]. Thirdly, osteocytes have several possible mechanosensors, including the cytoskeleton, osteocytes dendrites, focal adhesions, connexins, gap junctions, and ion channels. During OTM, osteocytes may activate other mechanosensors in response to mechanical forces to transduce the mechanical loading, thus affecting gene expression and regulating orthodontic bone remodeling [3,45,46,47]. 

Previous animal studies reported the effect of primary cilia in bone remodeling with universal deletion models and osteoblast- and osteocyte-specific deletion models [22,48,49,50,51]. Pkd1^m1Bei/m1Bei^ mice with a universal deletion of the Pkd1 gene, which encodes polycystin-1 (PC1), showed delayed intramembranous and endochondral osteogenesis through Runx2 inhibition [22]. Pkd1^+/m1bei^ mice presented with decreased bone mineral density, mineral apposition rate, and osteoblast/osteoclast marker expression, including osteocalcin, TRAP, OPG, and RANKL. Lehti et al. found that the cilia-related sperm flagellar protein 2 (SPEF2) regulates osteoblast differentiation using Spef2 KO mouse models [48]. The Spef2 KO mice presented shorter long bones and reduced bone mineral density, in comparison to the wild-type. Others observed the Pkd1^Dmp1-cKO^ mice, in which Pkd1 was conditionally deleted in mature osteoblasts and osteocytes [52]. Similar to our study, they did not find any skeletal abnormalities in Pkd1^Dmp1-cKO^ mice. However, the mechanical loading-induced anabolic bone response was hugely impaired in Pkd1^Dmp1-cKO^ mice, compared with wild-type mice, demonstrating that PC1 is a key mechanosensor in the anabolic reaction to mechanical loading in osteoblasts and osteocytes. Mature mice with a universal knockout of adenylyl cyclase 6 (AC6) showed normal bony phenotype, but with a 41% lower bone formation rate from mechanical loading to ulna, when compared to the control group [49]. Moreover, Cola1(I) 2.3-Cre.Kif3a^fl/fl^ mice, with an earlier stage osteoblast/osteocyte-specific deletion of Kif3a, presented no skeletal abnormalities but had a decreased response towards mechanical ulnar loading, in comparison to wild-type mice. In our study, both DMP1 CRE^+^.IFT80^f/f^ and DMP1 CRE^−^.IFT80^f/f^ mice responded similarly to orthodontic loading in the short-term and masticatory force in the long-term. Different responses might stem from the different cre mice, loading system used (both sides compression vs one side compression), anatomic site (long bones such as ulna and tibia vs maxilla), and length of force application (120 cycles/day for 3 consecutive days vs. 35 g of mechanical loading for 5 and 12 days).

To date, the role of cilia in OTM has rarely been examined. OTM comprises both compression and tension sides. Until now, many mechanobiology studies have focused on the cell responses to the stretch-induced tensile force or fluid shear stress [53,54]. In vitro studies demonstrate that fluid flow changes activate various cells through primary cilia. During mechanical loading, osteocytes produce various signals, such as NO, ATP, PGE_2_, and Ca2^+^, thereby stimulating bone remodeling [55]. Fluid flow increased the expression of the PGE_2_, COX2 mRNA, and OPG/RANKL mRNA ratio in MLO-Y4 osteocyte-like cells, whereas the cells treated with chloral hydrate or siRNA to remove cilia did not show significant changes [21]. Blocking primary cilia formation in osteoblasts and osteocytes interferes with the expression of osteogenic and osteoclastic cytokines and decreases their response to fluid flow changes [15]. Lineage-specific deletion of ciliary proteins, such as Kif3a, IFT20, IFT80, IFT88, and PC1, in osteoblasts or osteoblast precursors causes a lack of cilia formation, defective osteoblast differentiation, new bone formation, and mineralization when subjected to mechanical loading, suggesting an important effect of cilia in the bone-forming function on tension side during OTM [24,51,56]. The effect of conditional deletion of PC1 in the craniofacial region has been examined under orthodontic loading using PC1/Wnt1-cre mutant mice [57]. The Wnt1 promoter is highly expressed in the cranial neural crest cells, which have multipotent developmental potential and can generate multiple cell types, such as bones, cartilage, endocrine cells, and the peripheral nervous system. A calcium channel complex, which comprises the PC1 and PC2, is located at the base of the primary cilium and affects the cilia bending [58]. The authors noted a change in osteoclast activity, associated with PC1 deficiency, in PC1/Wnt1-cre mutant mice, followed by impaired OTM, which was possibly related to the absence of signal from the PDL. In addition to different target genes (PC1 vs IFT80), the authors examined OTM in cranial neural crest-derived cells, and we tested the OTM in late-stage mature osteocytes. In our study, both the experimental DMP1 CRE^+^.IFT80^f/f^ and control DMP1 CRE^−^.IFT80^f/f^ mice showed an increase in osteoclast formation and RANKL expression on the compression side during OTM. However, the differences between the experimental and control groups were not statistically significant, suggesting the limited effect of IFT80 deletion in osteocytes in vivo.

The role of osteocytes in bone remodeling has been studied extensively as osteocytes affect both osteoblasts and osteoclasts. Osteocytes have a number of mechanosensors, in which, we tested the possible role of primary cilia during OTM. Based on our findings, we can consider other mechanosensors in osteocytes for future bone remodeling studies. In addition, quite different conditions between in vivo and in vitro environments stress the importance of in vivo studies. 

To our knowledge, this study provides a novel examination on the effect of IFT80 deletion in osteocytes during OTM and physiologic bone remodeling in vivo. In response to mechanical loading, we observed no significant difference between the experimental DMP1 CRE^+^.IFT80^f/f^ and control DMP1 CRE^−^.IFT80^f/f^ mice in bone remodeling in both the short- and long-term, as demonstrated by similar OTM distances, osteoclast numbers, bone parameters, and the expression of RANKL and sclerostin. These results imply the anatomical limitations of primary cilia in osteocytes in vivo and the presence of possible mechanosensors in osteocytes and various force systems in OTM.

## Data Availability

The data presented in this study are available on request.

## Supplementary Materials

The following supporting information can be downloaded at: https://www.mdpi.com/article/10.3390/life12081147/s1, Figure S1: Genotyping results for DMP1 CRE and IFT80.

## References

[1] Shu R., Bai D., Sheu T., He Y., Yang X., Xue C., He Y., Zhao M., Han X. (2017). Sclerostin Promotes Bone Remodeling in the Process of Tooth Movement. PLoS ONE.

[2] Schaffler M.B., Cheung W.Y., Majeska R., Kennedy O. (2014). Osteocytes: Master orchestrators of bone. Calcif. Tissue Int..

[3] Qin L., Liu W., Cao H., Xiao G. (2020). Molecular mechanosensors in osteocytes. Bone Res..

[4] Klein-Nulend J., Bakker A.D., Bacabac R.G., Vatsa A., Weinbaum S. (2013). Mechanosensation and transduction in osteocytes. Bone.

[5] Shoji-Matsunaga A., Ono T., Hayashi M., Takayanagi H., Moriyama K., Nakashima T. (2017). Osteocyte regulation of orthodontic force-mediated tooth movement via RANKL expression. Sci. Rep..

[6] Odagaki N., Ishihara Y., Wang Z., Ei Hsu Hlaing E., Nakamura M., Hoshijima M., Hayano S., Kawanabe N., Kamioka H. (2018). Role of Osteocyte-PDL Crosstalk in Tooth Movement via SOST/Sclerostin. J. Dent. Res..

[7] Xiong J., Onal M., Jilka R.L., Weinstein R.S., Manolagas S.C., O’Brien C.A. (2011). Matrix-embedded cells control osteoclast formation. Nat. Med..

[8] Yamaguchi M. (2009). RANK/RANKL/OPG during orthodontic tooth movement. Orthod. Craniofac. Res..

[9] van Bezooijen R.L., ten Dijke P., Papapoulos S.E., Lowik C.W. (2005). SOST/sclerostin, an osteocyte-derived negative regulator of bone formation. Cytokine Growth Factor Rev..

[10] van Bezooijen R.L., Roelen B.A., Visser A., van der Wee-Pals L., de Wilt E., Karperien M., Hamersma H., Papapoulos S.E., ten Dijke P., Lowik C.W. (2004). Sclerostin is an osteocyte-expressed negative regulator of bone formation, but not a classical BMP antagonist. J. Exp. Med..

[11] Lee K.L., Guevarra M.D., Nguyen A.M., Chua M.C., Wang Y., Jacobs C.R. (2015). The primary cilium functions as a mechanical and calcium signaling nexus. Cilia.

[12] Hoey D.A., Chen J.C., Jacobs C.R. (2012). The primary cilium as a novel extracellular sensor in bone. Front. Endocrinol..

[13] Veland I.R., Awan A., Pedersen L.B., Yoder B.K., Christensen S.T. (2009). Primary cilia and signaling pathways in mammalian development, health and disease. Nephron Physiol..

[14] DeRouen M.C., Oro A.E. (2009). The primary cilium: A small yet mighty organelle. J. Investig. Dermatol..

[15] Whitfield J.F. (2003). Primary cilium—Is it an osteocyte’s strain-sensing flowmeter?. J. Cell Biochem..

[16] Yuan X., Serra R.A., Yang S. (2015). Function and regulation of primary cilia and intraflagellar transport proteins in the skeleton. Ann. N. Y. Acad. Sci..

[17] Kwon R.Y., Temiyasathit S., Tummala P., Quah C.C., Jacobs C.R. (2010). Primary cilium-dependent mechanosensing is mediated by adenylyl cyclase 6 and cyclic AMP in bone cells. FASEB J..

[18] Nguyen A.M., Jacobs C.R. (2013). Emerging role of primary cilia as mechanosensors in osteocytes. Bone.

[19] Norris D.P., Jackson P.K. (2016). Cell biology: Calcium contradictions in cilia. Nature.

[20] Reiter J.F., Leroux M.R. (2017). Genes and molecular pathways underpinning ciliopathies. Nat. Rev. Mol. Cell Biol..

[21] Malone A.M., Anderson C.T., Tummala P., Kwon R.Y., Johnston T.R., Stearns T., Jacobs C.R. (2007). Primary cilia mediate mechanosensing in bone cells by a calcium-independent mechanism. Proc. Natl. Acad. Sci. USA.

[22] Xiao Z., Zhang S., Mahlios J., Zhou G., Magenheimer B.S., Guo D., Dallas S.L., Maser R., Calvet J.P., Bonewald L. (2006). Cilia-like structures and polycystin-1 in osteoblasts/osteocytes and associated abnormalities in skeletogenesis and Runx2 expression. J. Biol. Chem..

[23] Ding D., Yang X., Luan H.Q., Wu X.T., He C., Sun L.W., Fan Y.B. (2020). Pharmacological Regulation of Primary Cilium Formation Affects the Mechanosensitivity of Osteocytes. Calcif. Tissue Int..

[24] Yang S., Wang C. (2012). The intraflagellar transport protein IFT80 is required for cilia formation and osteogenesis. Bone.

[25] Huang W., Kane J.K., Li M.D. (2008). Identification and characterization of a long isoform of human IFT80, IFT80-L. Biochem. Biophys. Res. Commun..

[26] Dallas S.L., Prideaux M., Bonewald L.F. (2013). The osteocyte: An endocrine cell... and more. Endocr. Rev..

[27] Lu Y., Xie Y., Zhang S., Dusevich V., Bonewald L.F., Feng J.Q. (2007). DMP1-targeted Cre expression in odontoblasts and osteocytes. J. Dent. Res..

[28] Feng J.Q., Ward L.M., Liu S., Lu Y., Xie Y., Yuan B., Yu X., Rauch F., Davis S.I., Zhang S. (2006). Loss of DMP1 causes rickets and osteomalacia and identifies a role for osteocytes in mineral metabolism. Nat. Genet..

[29] Yuan X., Cao J., He X., Serra R., Qu J., Cao X., Yang S. (2016). Ciliary IFT80 balances canonical versus non-canonical hedgehog signalling for osteoblast differentiation. Nat. Commun..

[30] Yang C.Y., Jeon H.H., Alshabab A., Lee Y.J., Chung C.H., Graves D.T. (2018). RANKL deletion in periodontal ligament and bone lining cells blocks orthodontic tooth movement. Int. J. Oral Sci..

[31] Jeon H.H., Yang C.Y., Shin M.K., Wang J., Patel J.H., Chung C.H., Graves D.T. (2021). Osteoblast lineage cells and periodontal ligament fibroblasts regulate orthodontic tooth movement that is dependent on Nuclear Factor-kappa B (NF-kB) activation. Angle Orthod..

[32] Andrade I., Taddei S.R., Garlet G.P., Garlet T.P., Teixeira A.L., Silva T.A., Teixeira M.M. (2009). CCR5 down-regulates osteoclast function in orthodontic tooth movement. J. Dent. Res..

[33] Andrade I., Silva T.A., Silva G.A., Teixeira A.L., Teixeira M.M. (2007). The role of tumor necrosis factor receptor type 1 in orthodontic tooth movement. J. Dent. Res..

[34] Taddei S.R., Moura A.P., Andrade I., Garlet G.P., Garlet T.P., Teixeira M.M., da Silva T.A. (2012). Experimental model of tooth movement in mice: A standardized protocol for studying bone remodeling under compression and tensile strains. J. Biomech..

[35] Bouxsein M.L., Boyd S.K., Christiansen B.A., Guldberg R.E., Jepsen K.J., Muller R. (2010). Guidelines for assessment of bone microstructure in rodents using micro-computed tomography. J. Bone Miner. Res..

[36] Tsutsumi T., Kajiya H., Tsuzuki T., Goto K.T., Okabe K., Takahashi Y. (2018). Micro-computed tomography for evaluating alveolar bone resorption induced by hyperocclusion. J. Prosthodont. Res..

[37] Shimizu Y., Ishida T., Hosomichi J., Kaneko S., Hatano K., Ono T. (2013). Soft diet causes greater alveolar osteopenia in the mandible than in the maxilla. Arch. Oral Biol..

[38] Sugawara Y., Kamioka H., Honjo T., Tezuka K., Takano-Yamamoto T. (2005). Three-dimensional reconstruction of chick calvarial osteocytes and their cell processes using confocal microscopy. Bone.

[39] Tanaka-Kamioka K., Kamioka H., Ris H., Lim S.S. (1998). Osteocyte shape is dependent on actin filaments and osteocyte processes are unique actin-rich projections. J. Bone Miner. Res..

[40] Wassermann F., Yaeger J.A. (1965). Fine structure of the osteocyte capsule and of the wall of the lacunae in bone. Z. Für Zellforsch..

[41] McNamara L.M., Majeska R.J., Weinbaum S., Friedrich V., Schaffler M.B. (2009). Attachment of osteocyte cell processes to the bone matrix. Anat. Rec..

[42] Coughlin T.R., Voisin M., Schaffler M.B., Niebur G.L., McNamara L.M. (2015). Primary cilia exist in a small fraction of cells in trabecular bone and marrow. Calcif. Tissue Int..

[43] Adachi T., Aonuma Y., Tanaka M., Hojo M., Takano-Yamamoto T., Kamioka H. (2009). Calcium response in single osteocytes to locally applied mechanical stimulus: Differences in cell process and cell body. J. Biomech..

[44] Klein-Nulend J., van der Plas A., Semeins C.M., Ajubi N.E., Frangos J.A., Nijweide P.J., Burger E.H. (1995). Sensitivity of osteocytes to biomechanical stress in vitro. FASEB J..

[45] Jeon H.H., Teixeira H., Tsai A. (2021). Mechanistic Insight into Orthodontic Tooth Movement Based on Animal Studies: A Critical Review. J. Clin. Med..

[46] Uda Y., Azab E., Sun N., Shi C., Pajevic P.D. (2017). Osteocyte Mechanobiology. Curr. Osteoporos. Rep..

[47] Li X., Kordsmeier J., Xiong J. (2021). New Advances in Osteocyte Mechanotransduction. Curr. Osteoporos. Rep..

[48] Lehti M.S., Henriksson H., Rummukainen P., Wang F., Uusitalo-Kylmala L., Kiviranta R., Heino T.J., Kotaja N., Sironen A. (2018). Cilia-related protein SPEF2 regulates osteoblast differentiation. Sci. Rep..

[49] Lee K.L., Hoey D.A., Spasic M., Tang T., Hammond H.K., Jacobs C.R. (2014). Adenylyl cyclase 6 mediates loading-induced bone adaptation in vivo. FASEB J..

[50] Temiyasathit S., Tang W.J., Leucht P., Anderson C.T., Monica S.D., Castillo A.B., Helms J.A., Stearns T., Jacobs C.R. (2012). Mechanosensing by the primary cilium: Deletion of Kif3A reduces bone formation due to loading. PLoS ONE.

[51] Qiu N., Xiao Z., Cao L., Buechel M.M., David V., Roan E., Quarles L.D. (2012). Disruption of Kif3a in osteoblasts results in defective bone formation and osteopenia. J. Cell Sci..

[52] Xiao Z., Dallas M., Qiu N., Nicolella D., Cao L., Johnson M., Bonewald L., Quarles L.D. (2011). Conditional deletion of Pkd1 in osteocytes disrupts skeletal mechanosensing in mice. FASEB J..

[53] Jufri N.F., Mohamedali A., Avolio A., Baker M.S. (2015). Mechanical stretch: Physiological and pathological implications for human vascular endothelial cells. Vasc. Cell.

[54] Booth R., Noh S., Kim H. (2014). A multiple-channel, multiple-assay platform for characterization of full-range shear stress effects on vascular endothelial cells. Lab. Chip.

[55] Temiyasathit S., Jacobs C.R. (2010). Osteocyte primary cilium and its role in bone mechanotransduction. Ann. N. Y. Acad. Sci..

[56] Yuan X., Yang S. (2016). Primary Cilia and Intraflagellar Transport Proteins in Bone and Cartilage. J. Dent. Res..

[57] Shalish M., Will L.A., Fukai N., Hou B., Olsen B.R. (2014). Role of polycystin-1 in bone remodeling: Orthodontic tooth movement study in mutant mice. Angle Orthod..

[58] Nauli S.M., Alenghat F.J., Luo Y., Williams E., Vassilev P., Li X., Elia A.E., Lu W., Brown E.M., Quinn S.J. (2003). Polycystins 1 and 2 mediate mechanosensation in the primary cilium of kidney cells. Nat. Genet..

