# Supplementary material for: The Effect of IFT80 Deficiency in Osteocytes on Orthodontic Loading-Induced and Physiologic Bone Remodeling: In Vivo Study
Source: Life (Basel). 2022 Jul 29;12(8):1147. doi: 10.3390/life12081147 (PMC9410307; doi:10.3390/life12081147)
Supplement: Supplementary file 1 [file life-12-01147-s001.zip › life-1799224-supplementary.pdf]

Supplementary Figure S1

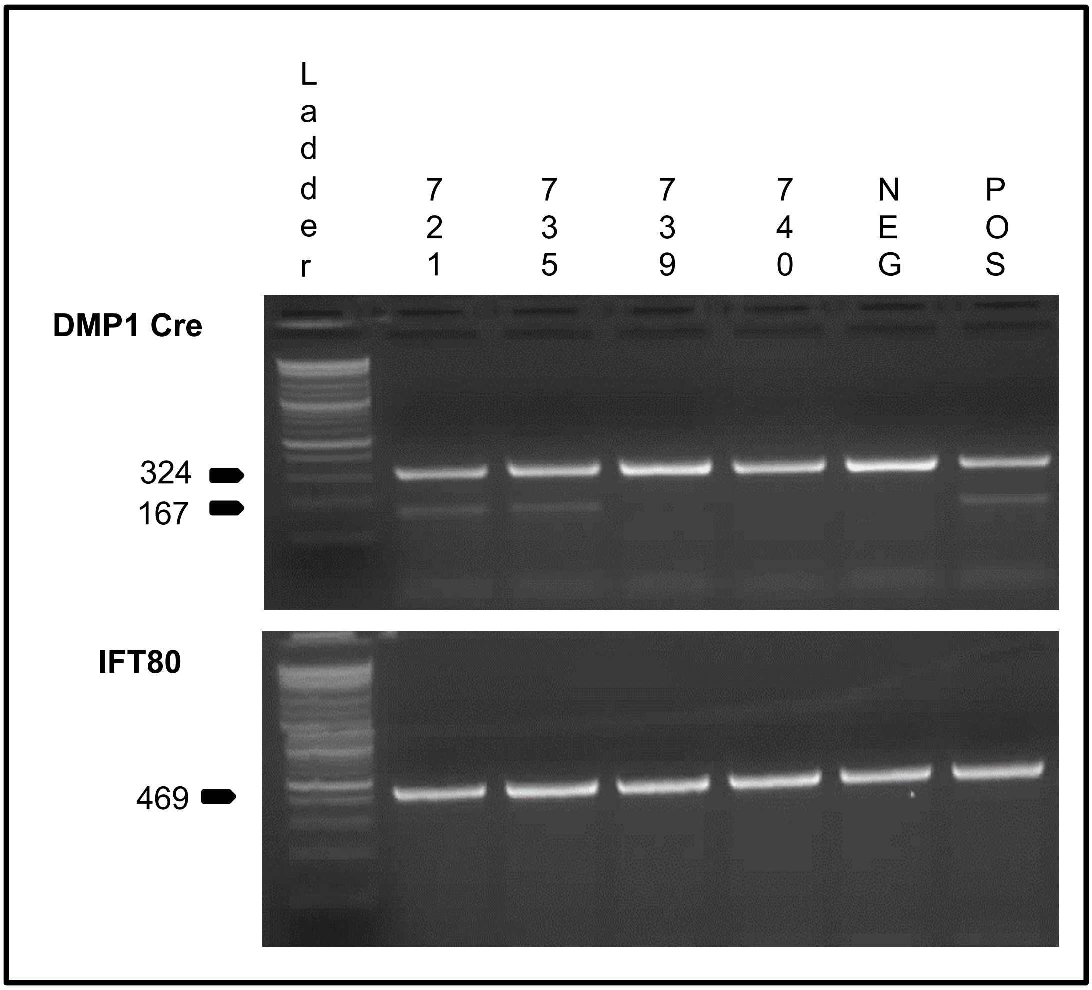

Supplementary Figure S1. Genotyping results for DMP1 Cre and IFT80. Genotyping was performed using the primers for DMP1-cre and IFT80 according to the manufacturer's instructions (Jackson Laboratory). [DMP1-cre] Transgene = 167bp and Internal positive control = 324 bp. [IFT80] Transgene = 469bp and WT =247 bp.
